# Potential of Agricultural Plantations and Orchards to Support Understory Forest Bird Diversity in Southeast Asia

**DOI:** 10.1002/ece3.73424

**Published:** 2026-04-10

**Authors:** Ku Noor Khalidah, Badrul Azhar, Natsuki Komada, Masumi Hisano, Tetsuro Hosaka

**Affiliations:** ^1^ Graduate School of Advanced Science and Engineering Hiroshima University Hiroshima Japan; ^2^ Department of Forest Science and Biodiversity, Faculty of Forestry and Environment University Putra Malaysia Serdang Malaysia

**Keywords:** forest reserves, land‐use change, matrix management, mist‐netting, oil palm, rubber tree, tropical plantations

## Abstract

Tropical forests hold two‐thirds of the world's biodiversity but have declined due to forest degradation and deforestation, mainly driven by agricultural expansion. Although agricultural lands, such as plantations and orchards, are matrices (i.e., unsuitable habitats) for many forest species in general, their impact on biodiversity could largely differ depending on the type of agricultural land and management practices. Therefore, understanding the impact of different agricultural land types on biodiversity is important for planning matrix management in tropical agricultural landscapes. This study assessed the potential of monoculture oil palm and rubber tree plantations, as well as polyculture orchards, to support understory bird diversity compared to forest reserves in Peninsular Malaysia using mist‐netting conducted between 2017–2023. Orchards recorded the highest bird abundance and richness among agricultural lands, but all agricultural lands had less than half the understory bird species found in forest reserves. Bird species composition also differed significantly among all habitats; forest specialists dominated (81.8%) in forest reserves, whereas nonforest specialists were dominant in orchards (76.3%), oil palm (100%), and rubber tree plantations (100%). Among habitat variables, understory vegetation covers positively affected bird species richness. Our results suggest that, despite having much lower diversity than forest reserves, polyculture orchards had some forest‐specialist species that possibly spilled over from forest reserves, and thus, are better habitats for understory bird communities than monoculture plantations. Maintaining crop diversity and understory vegetation complexity can improve matrix quality and mitigate the impact on biodiversity in landscapes dominated by monoculture plantations.

## Graphical abstract

This study assessed the potential of monoculture oil palm and rubber tree plantations, as well as polyculture orchards, to support understory bird diversity compared to forest reserves using mist‐netting. Orchards recorded the highest bird abundance and richness among agricultural lands, but all agricultural lands had less than half the understory bird species found in forest reserves.

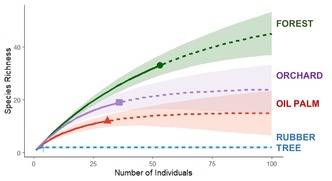

## 1 Introduction

Tropical forests, despite comprising only 10% of the Earth's land area, harbor over two‐thirds of global terrestrial biodiversity (Giam 2017; Bradshaw et al. 2009). Deforestation and degradation, primarily driven by agricultural expansion, threaten these ecosystems (Potapov et al. 2017; Achard et al. 2002). Global food demand is projected to double by 2050 from 2010 levels (Springmann et al. 2018), and currently about 30% of the world's net primary production is allocated for human use, with 38% of the global land surface dominated by agricultural land (Zabel et al. 2019; Ramankutty et al. 2008; Haberl et al. 2007). In tropical countries, agriculture expanded by approximately 48,000 km^2^ annually from 1999 to 2008 (Phalan et al. 2016). The production of tropical crops has increased rapidly since the 1960s, contributing to a net annual loss of more than 12 million ha of forest area (Arroyo‐Rodríguez et al. 2020; Watson et al. 2016; Hansen et al. 2013).

In Malaysia, one of the major biodiversity hotspots in the tropics (Myers et al. 2000), approximately 33.1% of the land area is allocated to agriculture, contributing 7.2% of the national GDP and accounting for 23% of total exports (Ministry of Primary Industry of Malaysia (MPIC) 2015; Rozhan 2015). Agricultural intensification, initiated during British colonial rule, introduced oil palm ( *Elaeis guineensis* ) and rubber tree ( *Hevea brasiliensis* ) plantations from South Africa and Brazil, respectively (Sayer et al. 2012; Nath and Chaudhuri 2010). Malaysia is now the second‐largest global palm oil producer, with 5.9 million ha of oil palm plantations and employing over 600,000 people (Fitzherbert et al. 2008). Malaysia was once the leading rubber producing country during the 1960s, but has declined to the seventh place, though rubber tree plantations still cover 1.02 million ha (Malaysian Rubber Board 2023; International Rubber Study Group 2022; MRB 2020; Barlow et al. 2014). Orchards, including durian and rambutan, occupy approximately 200,000 ha (Department of Agriculture Malaysia (DOA) 2023; Malaysian Agricultural Research and Development Institute (MARDI) 2022).

Loss due to the conversion of forests into agricultural land has severe consequences on global biodiversity, climate, and carbon dynamics (Duguma et al. 2019; Symes et al. 2018; Gibson et al. 2011). Conversion to agricultural lands has altered the species composition and decreased the population size of animal and plant species (Yahya et al. 2022; Green et al. 2020; Azhar et al. 2011), resulting in 30% of forest‐dependent species becoming vulnerable (IUCN 2019).

Plantations are regarded as unsuitable habitats for many forest‐dwelling species. However, some types of agricultural plantations can also serve as corridors, refuges, or alternative habitats if they provide resources such as foraging sites for these species (Perfecto et al. 2019). Among tropical farmlands, monoculture plantations with uniform stand structures, such as similar stand heights and trunk diameters resulting from identical planting ages, often reduce biodiversity more severely than polyculture farms (e.g., mixed orchards) (Azhar et al. 2015).

Matrix management is an approach for managing nonhabitat areas (e.g., agricultural lands) surrounding natural habitats (e.g., forests) to enhance the habitat quality of the landscape as a whole (Tscharntke et al. 2012). Matrix management includes promoting habitat heterogeneity, reducing the use of pesticides and fertilizers, and building ecological corridors for animals to reduce the negative effects of intensive agricultural practices on biodiversity. This approach enables us to implement more effective species conservation than relying solely on small and patchy natural habitats (Fahrig 2001). Therefore, understanding the differences in the impacts of different types of agricultural plantations on biodiversity is important for planning matrix management in tropical plantation landscapes (Zermeño‐Hernández et al. 2016).

Bird communities in tropical forests are vital for ecosystem services (Mohd‐Azlan et al. 2019; Sekercioglu 2012; Thinh 2006), and their sensitivity to forest structure is an excellent indicator of forest disturbance (Whelan et al. 2015). In particular, understory bird species are known to be microhabitat specialists, making them particularly sensitive to habitat changes (Bitani et al. 2023). Previous studies have shown that agricultural practices, including plantation cultivation, significantly alter bird communities (Pham Thi et al. 2021; Zeng et al. 2018; Drescher et al. 2016). A comparison of understory bird communities across different types of plantations can reveal the impact of land‐use changes on biodiversity (Edwards et al. 2014; Sheldon et al. 2010).

Yahya et al. (2022) reported that both overall bird abundance and species richness were significantly greater in agroforestry orchards than in monocultured oil palm and rubber tree plantations. These findings underscore the ecological value of orchard‐based agroforestry systems as important refuges for farmland bird communities in tropical agricultural plantations. However, our understanding of the extent to which tropical plantations and orchards can retain bird diversity compared to nearby forest reserves remains limited, especially in Southeast Asia, where direct comparisons are scarce. Hence, we addressed the following research questions:

- To what extent do plantations and orchards retain understory forest bird communities in terms of abundance and species richness and composition?
- Which habitat characteristics influence bird diversity across all habitat types?

As habitat characteristics, we focused on understory vegetation cover and height, mature tree abundance, elevation and proximity to forest. Understory vegetation cover and height and mature tree abundance may positively influence species richness due to increased foraging sites and refuges (Munian et al. 2023; Atikah et al. 2021). Proximity to forest may also positively affect species richness due to landscape spillover processes and dispersal limitations (Blitzer et al. 2012). Elevation was not a habitat characteristic of our interest in this study, but was included to control its effect because altitudinal variation can influence species richness and composition through vegetation and climatic differences (Santillán et al. 2020). Elucidation of the key habitat characteristics could provide useful information for better matrix management.

## 2 Materials and Methods

### 2.1 Research Area

We conducted this study in two forest reserves (Angsi Forest Reserve and Sungai Menyala Forest Reserve), two dominant plantations and orchards in the state of Negeri Sembilan, Peninsular Malaysia (Figure S1). All the study sites in plantations and orchards were located between the two forest reserves, at a distance of approximately 4–6 km from the nearest forest reserve.

Angsi Forest Reserve (2°41′54″ N, 102°02′53″ E) and Sungai Menyala Forest Reserve (2°29′7.76″N, 101°53′52.61″ E). The Angsi Forest Reserve is a permanent forest reserve that includes a continuous forest of 12,435 ha at 446–825 m above sea level (Faradiana et al. 2022). The forest is part of a linear corridor connected to the central forest spine in peninsular Malaysia (Faradiana et al. 2022; Haja‐Maideen et al. 2011). The vegetation of the Angsi Forest Reserve includes lowland and hill dipterocarp forests and secondary forests that regrew after logging from 1963 to 1964 (Hamzah et al. 2004). Sungai Menyala Forest Reserve has a 1280 ha area of unlogged forest (Hazwan et al. 2022) at 20–60 m above sea level (Samantha et al. 2020).

We selected traditional orchards cultivated with multiple native fruit tree species along with oil palm and rubber plantations as the study sites within the Rembau district (Figure S1). The rubber tree and oil palm plantations are owned and managed by small or large holding companies (either government‐linked or private) and planted with a monoculture practice using chemical herbicides and pesticides. Latex from rubber trees is typically collected daily, whereas oil palm fruit bunches are harvested twice a month. The ages of the oil palm and rubber trees at our study sites ranged from 11 to 20 and 15 to 30 years, respectively.

The fruit orchards were owned by local households and planted on 0.4–2.8 ha of land. Local owners manage orchards using polyculture farms and use organic pesticides (e.g., neem oil) and herbicides (e.g., salt and vinegar) instead of chemical pesticides. The crop species in the targeted orchards included rambutan ( *Nephelium lappaceum* ), durian ( *Durio zibethinus* ), mangosteen ( *Garcinia mangostana* ), jackfruit ( *Artocarpus heterophyllus* ), papaya ( *Carica papaya* ), langsat ( *Lansium domesticum* ), pineapple ( *Ananas comosus* ), and dragon fruits (*Selenicereus undatus*). They are typically collected twice a year during peak fruiting seasons.

### 2.2 Bird Survey

We used mist‐netting to evaluate understory bird diversity. Mist‐netting captures small, secretive species (Barlow et al. 2006; Wang and Finch 2002; Rappole et al. 1998) and allows precise habitat association (Maas et al. 2019; Randler and Kalb 2018; Brzorad and Maccarone 2014).

We conducted mist‐netting survey in plantations and orchards in 2017–2018, and in forest reserves in 2023. As far as we know, there were no major differences in climatic conditions and the management of plantations, orchards, and forests between the two periods. Mist nets were installed at 300 m intervals starting from random start points (Thomas et al. 2020; Morrison et al. 2008) (Figure S2). We installed meshed mist nets (size: 9 × 3 m) at 150 points in total: 40 each in the orchard, oil palm, and rubber plantations, while the remaining 30 points were installed in forest habitats, with 16 points in the Angsi Forest Reserve and 14 points in the Sungai Menyala Forest Reserve. We sampled the birds from dusk until dawn (0700–1900 h) for three consecutive days, with a total of 36 net hours for each point. We censused each location every 15–20 min to avoid stress to the captured birds caused by prolonged time in the net. We identified the captured birds as described by Jeyarajasingam (2012), cut their nails for individual identification, released them, and no individuals were ringed. We conducted surveys when the weather was calm and nonrainy, which is appropriate for species recognition and safe for bird capture (Mulvaney and Cherry 2020).

To compare the habitat requirements of bird species, we divided the species into two habitat guilds based on their reliance on forests: (1) forest specialists (species primarily found in forests) and (2) nonforest specialists (species not primarily found in forest areas) based on the literature and published data (Tohiran et al. 2019; BirdLife International 2014; Jeyarajasingam 2012). Because bird feeding guilds are related to their ecological functions (Tchoumbou et al. 2020); nectarivores contribute to pollination, granivores and frugivores to seed dispersal, and insectivores to pest control (Van Bael et al. 2007; Şekercioğlu et al. 2004), we also classified birds into feeding guilds based on their primary diets: (1) frugivores (fruit‐feeders), (2) carnivores (animal‐feeders), (3) insectivores (insect‐feeders), (4) piscivores (fish‐feeders), and (5) frugivore–insectivores (fruit and insect‐feeders), based on Jeyarajasingam (2012) in each habitat.

### 2.3 Measurement of Habitat Characteristics

We measured habitat characteristics within 20 × 20 m plots around each mist‐ netting point (Figure S2). The habitat characteristics measured were (1) understory vegetation cover, (2) understory vegetation height, (3) mature tree abundance, (4) elevation and (5) proximity to forest. We measured understory vegetation cover within 2 m^2^ quadrats placed five meters from each mist‐netting point in the east, west, north, and south based on photographs taken using a mobile phone with the *Canopeo* application (Schweizer et al. 2024). Understory vegetation height was the maximum height of the understory vegetation measured using measuring tapes in the same quadrats as reported (Harahap et al. 2019; Mohamed et al. 2012) and the mean was calculated. We defined tree abundance as the number of trees greater than 20 cm in Diameter at Breast Height (DBH), including planted trees and palms within a 20 m × 20 m plot (Bendall et al. 2024). This threshold is commonly used to represent mature trees that contribute disproportionately to forest vertical structure and fruit production, which could affect habitat quality for understory bird communities (Stagoll et al. 2012; Gil‐Tena et al. 2007). We recorded elevation at each point using a handheld Global Positioning System (GPS) unit and reported in meters above sea level (McCain 2009). We determined the distance to forests by measuring the distance between the mist‐netting points and the nearest forest reserve edge using the circular measurement function in Google Earth Pro (Razak et al. 2020). For forest plots, this distance was determined as 0 m.

### 2.4 Data Analysis

#### 2.4.1 Bird Species Richness Across Forests and Plantations

To ensure that detection biases among habitat types did not affect our results, we estimated sampling completeness by habitat type. We used Chao1 (Chao 1984), Jackknife1 (first‐order jackknife) (Bunge and Fitzpatrick 1993; Burnham and Overton 1979), Jackknife2 (second‐order jackknife) (Gotelli and Colwell 2011; Palmer 1991; Burnham and Overton 1978), and bootstrap estimators to estimate total species richness (Quan and Wang 2014; Wang et al. 2014) and evaluated sampling completeness with a sample size of 150. The standard errors for each estimator were also calculated to assess the variability associated with richness estimations. We also performed sample size‐based rarefaction species richness and extrapolated sampling curves on the data using the vegan package (Oksanen et al. 2013) in R version 4.2.3 (Team, R. C 2020).

We calculated Shannon diversity indices (*H′*) for understory bird communities in each habitat type to measure diversity by incorporating species richness and evenness. Higher *H′* values suggest increased diversity resulting from higher evenness, richness, or both (Magurran 2003; Shannon 1948). Diversity estimates were calculated individually for forest, orchard, oil palm, and rubber tree to allow comparison of community structure across different land uses. All analyses were performed in R using the vegan package (Oksanen et al. 2013).

#### 2.4.2 Bird Species Composition in Forests and Plantations

We used one‐way Analysis of Similarities (ANOSIM) to compare bird species composition among plantation, orchard, and forest habitats using Primer version 6 (Primer‐E Ltd.). The permutation test was repeated 999 times. To determine which specific pairs of groups showed significant differences, we applied post hoc tests with Holm–Bonferroni correction to account for multiple comparisons (Jafari and Ansari‐Pour 2018).

#### 2.4.3 Relationships Between Bird Species Richness and Habitat Characteristics

We constructed a generalized linear mixed model (GLMM) to predict bird species richness. The response variable was the number of bird species captured at each sampling point in the forest and plantation. Five habitat variables—(1) understory vegetation cover, (2) understory vegetation height, (3) mature tree abundance, (4) elevation (m), and (5) proximity to the forest (km)—were used as fixed variables after inspecting multicollinearity. We calculated the Pearson correlation coefficients between all pairs of the five habitat variables and variance inflation factors (VIFs) with the car package in R (Fox and Weisberg 2018). No serious multicollinearity was observed in both metrics; the maximum correlation coefficient among predictors was |*r*| = 0.64, which is below the commonly used threshold of |*r*| > 0.7 (Dormann et al. 2013) and none of the VIF values (understory vegetation growth = 1.32, understory vegetation height = 1.33, mature tree abundance = 1.74, proximity to the forest (km) = 3.09, elevation = 1.64) exceeded the thresholds (VIF < 5) (O'Brien 2007). Habitat types were also included as fixed variables to control confounding effects associated with different habitat types, with forest reserves used as the reference level. The Poisson distribution and log‐link functions were used in the GLMM. An overdispersion test based on Pearson residuals indicates only mild overdispersion (ĉ = 1.43) of the model, which is generally considered acceptable for Poisson GLMMs in ecological studies (Bolker et al. 2009). Therefore, a Poisson error distribution with a log‐link function was retained for the GLMM. Year and season were used as random variables. The seasons in this study were classified as summer (March–August) and winter (September–April) in temperate Asia (e.g., Japan and Korea) to account for bird migration between Southeast Asia and temperate Asia (Wei et al. 2024; BirdLife International 2014; Jeyarajasingam 2012).

## 3 Results

### 3.1 General Result

**FIGURE 1:**
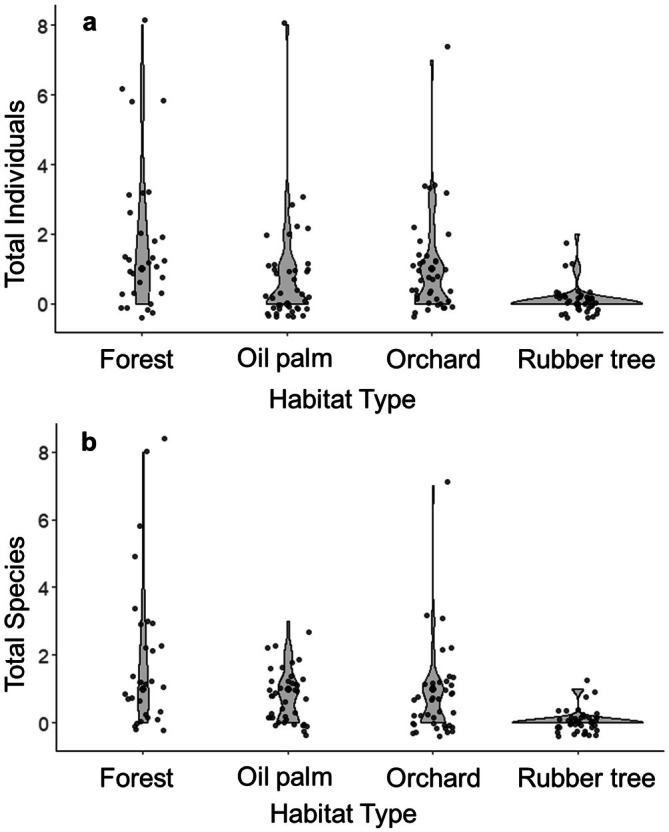
Number of understory bird species (a) and individuals (b) captured per mist‐net in four different habitat types.

In total, we recorded 128 individual birds belonging to 50 species (Table 1) from three agricultural plantations (orchard, oil palm, and rubber tree plantations) and forest reserves (Table S1). Forest reserves had the highest number of bird individuals and species, followed by orchards, oil palms, and rubber tree plantations. Similarly, the number of bird individuals and species per plot (Figure 1) was also the greatest in the forest (Mean per plot: 1.8 individuals and 1.8 species), followed by the orchard (1.0 and 0.8), oil palm (0.8 and 0.8), and rubber tree plantations (0.1 and 0.1) (Table 1).

**TABLE 1:** Bird species richness and abundance in all habitat types.

Habitat type	*N* of plots	*N* of species per plot (Mean ± SD)	*N* of individuals per plot (Mean ± SD)	Total species	Total individuals
Forest	30	1.8 ± 2.3	1.8 ± 2.1	33	54
Oil palm	40	0.8 ± 0.8	0.8 ± 1.5	14	32
Orchard	40	0.8 ± 1.3	1.0 ± 1.4	13	38
Rubber tree	40	0.1 ± 0.3	0.1 ± 0.4	2	4
Total	150			50	128

### 3.2 Estimates of Bird Species Richness in Four Different Habitats

**FIGURE 2:**
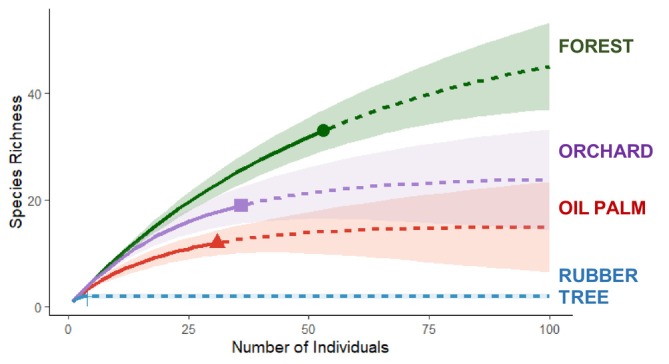
Sample size‐based rarefaction (solid lines) and extrapolation (dotted lines) sampling curves representing bird communities in four habitat types (oil palm and rubber plantations, orchards, and forest reserves).

The sampling completeness (%) ranged from 64.6% to 82.5% for orchards, 55.0% to 81.5% for oil palm, 66.7% to 80.3% for rubber, and 54.2% to 80.2% for forest reserves (Table 2). The similar range of sampling completeness values across all four study sites suggests that we detected a similar proportion of bird species among habitats. In terms of bird species richness, orchards, oil palm, and rubber plantations recorded 42%, 39%, and 6% of the species captured in the forest reserves, respectively (Figure 2).

**TABLE 2:** Estimated species richness and sampling completeness.

Land use	Observed Species	Chao	obs/Chao (%)	Jack1	jack1 (%)	Jack2	jack2 (%)	Boot	Boot (%)	*n*
All	51	76.9	66.4	75.8	67.3	88.7	57.5	62.1	82.2	150
Orchard	14	18.0	77.6	20.1	69.8	21.7	64.6	17.0	82.5	40
Oil palm	13	20.9	62.0	19.8	65.6	23.7	54.9	16.0	81.5	40
Rubber tree	2	2.5	80.4	2.9	67.2	3.00	66.7	2.5	80.3	40
Forest	33	52.4	62.9	51.4	64.2	60.9	54.2	41.1	80.2	30

Shannon diversity (*H′*) was the highest in oil palm plantations (*H′* = 3.06), followed by orchards (*H′* = 2.80) and forests (*H′* = 2.75), whereas rubber tree plantations supported the lowest diversity (*H′* = 1.10) (Table S2).

### 3.3 Bird Species Composition in Four Different Habitats

**FIGURE 3:**
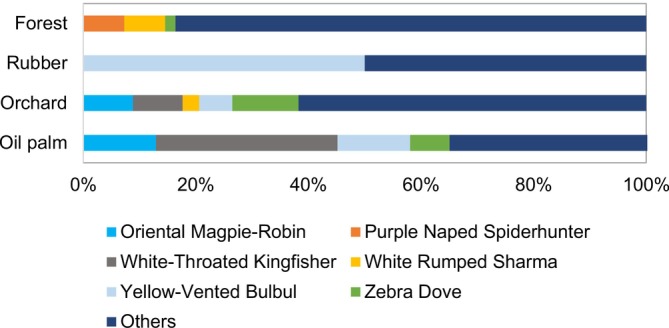
Bar chart of the relative abundance (%) of the most commonly recorded bird species in four different habitat types.

Bird species composition differed significantly across plantations, orchards, and forest reserves (ANOSIM, Global *R* = 0.05, *p <* 001) (Table 3, Figure 3). Post hoc analysis confirmed that bird species composition differed significantly between forest reserves and each plantation, and even among plantations, except between oil palm plantations and orchards.

**TABLE 3:** Comparison of bird species composition among orchards, oil palm, and rubber tree plantations and forest reserves based on one‐way analysis of similarities (ANOSIM).

Groups	*R*	*p* value
Oil Palm, Rubber Tree	0.071	0.001
Oil Palm, Orchard	0.011	0.112
Oil Palm, Forest	0.087	0.001
Rubber Tree, Orchard	0.022	0.004
Rubber Tree, Forest	0.062	0.001
Orchard, Forest	0.051	0.001

**FIGURE 4:**
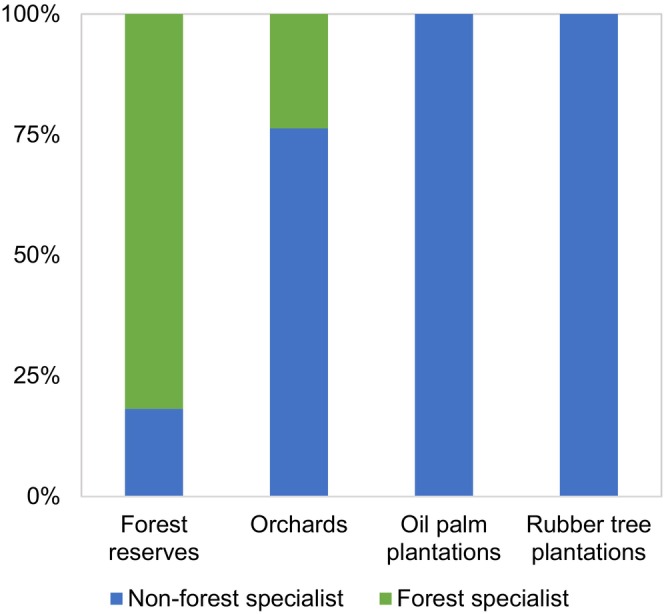
Comparison between number of forest and nonforest bird specialists in four different habitat types (oil palm and rubber plantations, orchards, and forest reserves).

Most birds were forest specialists (81.8%) in the forest reserves, whereas most birds (76.3%) were nonforest specialists in orchards. No forest specialists were observed in oil palm or rubber plantations (Figure 4). We captured four feeding guilds in the forest reserves (Figure S3): frugivore–insectivores, insectivore, frugivore, and piscivore. Although we recorded one more feeding guild, carnivore, in orchards, we only recorded three and one feeding guild in oil palm and rubber plantations, respectively.

### 3.4 Relationship Between Understory Bird Species and Habitat Characteristics

After controlling for the effect of land‐use type, understory vegetation cover had a significant impact on the number of understory bird species per trap (Table 4). The number of understory bird species increased as understory vegetation cover increased. In contrast, understory vegetation height, mature tree abundance, elevation, and proximity to the forest had no statistically significant effects on the number of understory bird species. Again, the significantly higher number of understory birds in forests than in orchards and plantations was also indicated by GLMM (Table 4).

**TABLE 4:** Coefficients of habitat characteristics based on generalized linear mixed model (GLMM) to predict understory bird species richness (number of species per plot). Forest reserve was set as the reference level for the habitat type. PTF (km) refers to the proximity to the nearest forest reserve, measured in kilometers.

	Estimate	Standard Error	*z* value	Pr(>|*z*|)
Understory vegetation cover	0.015	0.005	2.924	0.003
Understory vegetation height	−0.003	0.005	−0.720	0.471
Mature tree abundance	0.131	0.032	0.410	0.682
Elevation	−0.001	0.001	−0.749	0.454
PTF (km)	0.477	0.272	1.755	0.079
Habitat type_ oil palm	−3.667	1.356	−2.704	0.006
Habitat type_orchard	−3.765	1.458	−2.582	0.009
Habitat type_ rubber tree	−5.981	1.463	−4.089	< 0.001

Limited overlap occurred mainly in orchards, where a few forest‐associated species (e.g., White‐rumped Shama) were recorded at low abundances. Oil palm plantations showed minor overlap involving habitat‐tolerant species (e.g., Oriental Magpie‐Robin), while rubber tree plantations shared almost no species with forest. Overall, these patterns indicate weak and spatially restricted biodiversity spillover from forest into agricultural plantations.

## 4 Discussion

### 4.1 Diversity and Composition of Understory Birds

Species richness and composition of understory birds differed greatly between plantations/orchards and forest reserves. Orchards and plantations had less than half the bird species compared to forests, which were dominated by nonforest specialists. This highlights the fact that agricultural plantations are not ecologically comparable to forest reserves in supporting diverse and specialized bird communities.

The alternation of bird species from habitat specialists to more widespread and common species in agricultural plantations has also been reported in other studies (e.g., Guerrero et al. 2024; Sekercioglu 2012). The reduced diversity and abundance of understory birds in plantations and orchards compared to those in forest reserves could be due to reduced habitat complexity (Yahya et al. 2022; Demestihas et al. 2017; Horak et al. 2013), less diverse or less abundant food sources (Sritongchuay et al. 2016), and frequent human activities (Rigal et al. 2023). Our results indicate that forest reserves are irreplaceable habitats for forest specialists, as suggested in previous studies (Barlow et al. 2016; Gibson et al. 2011).

Polyculture orchards exhibited higher species richness than the monoculture oil palm and rubber plantations. These orchards have more diverse floristic compositions and more complex strata of two or more perennial fruit trees, and we found nine forest specialist species. Polyculture orchards could be key habitats for both forest specialists and many nonforest specialists (Yahya et al. 2022; Jayathilake et al. 2021) in landscapes dominated by monoculture plantations. Notably, the presence of these forest‐associated species in orchards may reflect limited biodiversity spillover from nearby forest reserves as several species recorded in orchards were also found in forests. This partial species overlap suggests that, when the habitat structure is sufficiently complex, polyculture orchards may function as spillover zones or ecological extensions of nearby forests, facilitating movement or temporary use by forest birds.

Oil palm plantations had a similar or slightly smaller number of species than orchards, but lacked forest specialist species. The simple vegetation structure and frequent use of agrochemicals, including herbicides and pesticides, may make these landscapes unfavorable for forest‐specialist species (Sánchez‐Bayo and Wyckhuys 2019). Industrial monocultures are typically managed under highly intensive regimes that rely heavily on agrochemicals, particularly herbicides, to suppress vegetation undergrowth (Tohiran et al. 2017). Such chemical dependency not only reduces habitat complexity, but may also create chemically inhospitable conditions that deter forest species from venturing into or persisting within these plantations, even when located nearby forest reserves. The absence of forest specialists and distinct species composition further suggests that oil palm and rubber plantations are largely resistant to biodiversity spillover from forests, especially in structurally simplified or chemically intensive settings. Our findings are consistent with those of Cardoso et al. (2025) who reported limited evidence of biodiversity spillover from forest fragments to oil palm plantations in the Amazon, underscoring the challenge of facilitating forest species persistence in monoculture‐dominated landscapes.

Rubber tree plantations had the smallest number of species and had no forest specialists. In addition to the uniform habitat structure and intensive use of agrochemicals, the lack of attractive fruits for frugivorous birds, more intensive undergrowth clearing, and more frequent human disturbances (e.g., latex tapping every morning) than in oil palm plantations make rubber plantations more unfavorable to most tropical bird species (Dayananda et al. 2024; Hua et al. 2022; Zhang et al. 2017; Ayat and Tata 2015). Rubber tree agroforestry approaches may support diverse bird species (Azhar et al. 2017). Beukema et al. (2007) found that rubber tree agroforests (forest‐like lands) supported bird diversity, including forest and non‐forest species, in degraded landscapes dominated by monoculture plantations. Therefore, rather than the crop type, the diversity and structural limited biodiversity spillover observed in our study aligns with these findings and reinforces the importance of improving habitat complexity to promote potential spillover effects.

A caveat of this study is that forest reserves were surveyed approximately 5 years later than orchards and monoculture plantations. As far as we know, however, there were no major changes in regional climatic conditions and the management of plantations, orchards, and forests during this period. Therefore, we believe it is unlikely that this temporal difference significantly affected our findings.

We should also note the strengths and limitations of mist‐netting sampling for bird diversity. Mist‐netting is particularly useful for detecting cryptic, small, and understory‐dependent bird species, which are often overlooked by acoustic surveys or point counts, especially in structurally complex habitats such as tropical forests (Mulvaney and Cherry 2020; Arizaga et al. 2011; Pagen et al. 2002).

On the other hand, it can underestimate highly mobile, canopy‐dwelling or vocal species, and capture probabilities may vary with net height and vegetation structure (Tattoni and LaBarbera 2023; Pagen et al. 2002). As a result, our study does not represent complete bird assemblages in forest reserves, plantations, and orchards. Mist‐netting combined with complementary methods such as point counts or acoustic monitoring would likely yield better estimates of total bird diversity.

### 4.2 Influence of Habitat Characteristics on Bird Species Richness

Among habitat variables, understory vegetation covers positively affected the number of understory bird species. Shrubs and understory provide breeding, foraging, and refuge sites for understory bird species (Sweeney et al. 2010; Gil‐Tena et al. 2007). Our findings suggest that promoting understory vegetation cover improves the habitat quality of understory bird species across both forest and plantation habitats.

In contrast, tree abundance and proximity to forests had no statistically significant effects on the number of understory bird species. Rather than tree abundance, the taxonomic and structural diversity (e.g., understory, multiple crown layers, deadwood) of trees might affect bird diversity more strongly (Stagoll et al. 2012). Almost all of our sampling points in the plantations and orchards were more than 4 km away from the forests (Figure S4). This may partly explain why proximity to forests had no significant effect and why most of the captured birds in the plantations and orchards were nonforest specialists. As some studies have shown positive effects of proximity to forests on biodiversity in cocoa and coffee plantations (Oon et al. 2023; Luskin and Potts 2011), future studies should include plantations and orchards closer to forest reserves to examine such a spillover effect.

### 4.3 Conservation Implications

Our results suggest that agricultural plantations and orchards support less than half of the understory bird species supported by forests. Our findings support the irreplaceable role of natural forests in biodiversity conservation, compared to plantations and agroforestry systems (Narayana et al. 2024; Gibson et al. 2011; Azhar et al. 2011). In contrast, the higher diversity observed in polyculture fruit orchards than in monoculture plantations highlights their potential role as refuges for understory birds, particularly in landscapes dominated by monoculture plantations.

Although the conservation value of polyculture orchards has often been overlooked, orchards with sustainable management practices that minimize the use of agrochemicals and preserve vegetation complexity can contribute to the maintenance of biodiversity and ecosystem services (e.g., pest control) in agricultural plantations by serving as valuable stepping‐stone corridors and refuges for understory birds (Tohiran et al. 2024; Yahya et al. 2024; Denan et al. 2020). The occurrence of forest specialists in polyculture orchards suggests a limited but ecologically relevant biodiversity spillover from forest reserves, particularly when landscape elements such as vegetative complexity are retained. This finding supports the notion that structurally complex agricultural matrices can partially extend the conservation value of nearby forests, even when located several kilometers away. However, consistent with the findings of Cardoso et al. (2025), the absence of forest specialists in structurally simplified monocultures, such as oil palm plantations, indicates that, without significant habitat improvements, the potential for biodiversity spillover remains minimal.

Promoting the adoption of sustainable orchard management practices can help mitigate the negative effects of agricultural expansion on understory bird populations and contribute to the conservation of biodiversity in human‐dominated landscapes. Although previous studies have primarily focused on the irreplaceable role of natural forests, it is important to consider appropriate matrix management around forest reserves under the rapid expansion of tropical plantations. There is a need for a more nuanced approach to biodiversity conservation that recognizes the potential of diverse agricultural plantations to support biodiversity and contribute to ecosystem resilience, including mechanisms such as biodiversity spillover where conditions are favorable.

### 4.4 Protection of Human Subjects and Animals in Research

All procedures involving animals were evaluated and approved by the appropriate institutional review committee. Mist‐netting and field sampling of birds followed institutional and national rules for animal care in research. All precautions were taken to reduce stress and harm to the birds during capture, handling, and release.

## Author Contributions

**Ku Noor Khalidah:** data curation (lead), formal analysis (lead), investigation (lead), visualization (lead), writing – original draft (lead). **Badrul Azhar:** conceptualization (lead), data curation (equal), methodology (equal), resources (equal), writing – review and editing (equal). **Natsuki Komada:** formal analysis (equal), writing – review and editing (equal). **Masumi Hisano:** formal analysis (equal), writing – review and editing (equal). **Tetsuro Hosaka:** funding acquisition (lead), supervision (lead), validation (supporting), writing – review and editing (equal).

## Funding

This work was partly supported by a JSPS KAKENHI grant (grant number 23KK0197) and a JASTE Young Researcher Grant awarded to K. N. K.

## Conflicts of Interest

The authors declare no conflicts of interest.

## Supporting information


**Table S1:** List of bird species and number of individual birds in four different landscapes. Habitat type is based on literature and published data (Razak et al. 2020; Azman et al. 2019; Tohiran et al. 2019; BirdLife International 2014; Jeyarajasingam, A., 2012).
**Table S2:** Shannon diversity (H′) of understory birds across four habitat types. H′ accounts for both species richness and evenness, with higher values indicating greater diversity.
**Figure S1:** Map of the sampling location in plantations and forest reserves in Negeri Sembilan, Malaysia (Zoom‐out (a) and zoom‐in (b)).
**Figure S2:** Schematic layout of mist‐net in the sampling point location within 20 m x 20 m plot (trees shown in diagram are for illustration purposes only; actual tree locations varied).
**Figure S3:** Percentage of bird feeding guild in four different landscapes; Orchards, oil palms, rubber tree plantations and forest.
**Figure S4:** Violin plot of (a) understory vegetation cover (%), (b) understory vegetation height (cm), (c) mature tree abundance, (d) elevation (m), (e) proximity to forest (km) for each habitat type.

## Data Availability

The data and R code supporting the findings of this study are openly available in Figshare at https://doi.org/10.6084/m9.figshare.30911903. The repository contains all variables used in the analyses and the related scripts necessary to reproduce the results presented in the manuscript. These codes and data were made available to editors and reviewers during the peer‐review process and will be publicly accessible upon acceptance.
