# Supplementary material for: Potential of Agricultural Plantations and Orchards to Support Understory Forest Bird Diversity in Southeast Asia
Source: Ecol Evol. 2026 Apr 10;16(4):e73424. doi: 10.1002/ece3.73424 (PMC13066770; doi:10.1002/ece3.73424)
Supplement: Supplementary file 1 — Table S1: List of bird species and number of individual birds in four different landscapes. Habitat type is based on literature and published data (Razak et al. 2020; Azman et al. 2019; Tohiran et al. 2019; BirdLife International 2014; Jeyarajasingam, A., 2012). Table S2: Shannon diversity (H′) of understory birds across four habitat types. H′ accounts for both species richness and evenness, with higher values indicating greater diversity. Figure S1: Map of the sampling location in plantations and forest reserves in Negeri Sembilan, Malaysia (Zoom‐out (a) and zoom‐in (b)). Figure S2: Schematic layout of mist‐net in the sampling point location within 20 m x 20 m plot (trees shown in diagram are for illustration purposes only; actual tree locations varied). Figure S3: Percentage of bird feeding guild in four different landscapes; Orchards, oil palms, rubber tree plantations and forest. Figure S4: Violin plot of (a) understory vegetation cover (%), (b) understory vegetation height (cm), (c) mature tree abundance, (d) elevation (m), (e) proximity to forest (km) for each habitat type. [file ECE3-16-e73424-s001.docx]

**Appendices**

Table S1: List of bird species and number of individual birds in four different landscapes. Habitat type is based on literature and published data (Razak et al., 2020; Azman et al., 2019; Tohiran et al., 2019; BirdLife International, 2014; Jeyarajasingam, A., 2012)

| Scientific Name | Common name | Habitat type | Feeding guild | Oil palm | Rubber | Orchard | Forest |
| --- | --- | --- | --- | --- | --- | --- | --- |
| *Chalcophaps indica* (Linnaeus, 1758) | Asian Emerald Dove | Forest | Granivore,  frugivore | 1 | 0 | 0 | 0 |
| *Irena puella*  (Latham, 1790) | Asian Fairy Bluebird | Forest | Frugivore | 0 | 0 | 0 | 1 |
| *Aplonis panayensis*  (Scopoli, 1786) | Asian Glossy Starling | Forest, | Frugivore,  insectivore | 0 | 0 | 3 | 1 |
| *Ploceus philippinus*  (Linnaeus, 1766) | Baya Weaver | Forest, Shrubland, Grassland, | Granivore,  insectivore | 2 | 0 | 0 | 0 |
| *Pycnonotus atriceps*  (Temminck, 1822) | Black-headed Bulbul | Forest, Shrubland, Grassland, | Frugivore,  insectivore | 0 | 0 | 0 | 3 |
| *Oriolus chinensis*  (Linnaeus, 1766) | Black-naped Oriole | Forest, | Frugivore,  insectivore | 1 | 0 | 0 | 0 |
| *Microhierax fringillarius*  (Drapiez, 1824) | Black-thighed Falconet | Forest | Insectivore | 0 | 0 | 3 | 0 |
| *Alcedo euryzona*  (Temminck, 1830) | Blue-banded Kingfisher | Forest | Piscivore | 0 | 0 | 0 | 1 |
| *Alcedo meninting*  (Horsfield, 1821) | Blue-eared Kingfisher | Forest, Wetlands (inland) | Piscivore, carnivore | 0 | 0 | 2 | 2 |
| *Merops viridis*  (Linnaeus, 1758) | Blue-throated Bee-Eater | Forest, Shrubland, Wetlands | Insectivore | 1 | 0 | 0 | 0 |
| *Lanius cristatus*  (Linnaeus, 1758) | Brown Shrike | Forest, Shrubland, Grassland, Desert, | Carnivore, insectivore | 0 | 0 | 2 | 0 |
| *Iole olivacea*  (Blyth, 1844) | Buff-vented Bulbul | Forest, | Omnivore | 0 | 0 | 0 | 1 |
| *Enicurus ruficapillus*  (Temminck, 1832) | Chestnut-naped Forktail | Forest | Insectivore | 0 | 0 | 0 | 2 |
| *Ardeola bacchus*  (Bonaparte, 1855) | Chinese Pond Heron | Forest, Grassland, Wetlands | Carnivore | 1 | 0 | 0 | 0 |
| *Chalcophaps indica*  (Linnaeus, 1758) | Common Emerald Dove | Forest, | Granivore,  frugivore | 0 | 0 | 0 | 2 |
| *Acridotheres tristis*  (Linnaeus, 1766) | Common Myna | Forest, Grassland, | Omnivore | 2 | 0 | 1 | 0 |
| *Aethopyga siparaja*  (Raffles, 1822) | Crimson Sunbird | Forest, Shrubland, Grassland, | Omnivore | 0 | 0 | 0 | 1 |
| *Dicrurus annectans*  (Hodgson, 1836) | Crow Billed Drongo | Forest, Savanna, Shrubland, | Insectivore | 0 | 0 | 0 | 1 |
| *Arachnothera affinis*  (Eyton, 1839) | Grey-breasted Spiderhunter | Forest, Shrubland, | Nectarivore | 0 | 0 | 0 | 1 |
| *Stachyris poliocephala*  (Temminck, 1836) | Grey-headed Babbler | Forest, | Insectivore | 0 | 0 | 0 | 1 |
| *Tricholestes criniger*  (Blyth, 1845) | Hairy-backed Bulbul | Forest | Frugivore,  insectivore | 0 | 0 | 0 | 1 |
| *Cyornis banyumas*  (Horsfield, 1821) | Hill Blue Flycatcher | Forest, | Insectivore | 0 | 0 | 0 | 2 |
| *Acridotheres javanicus*  (Cabanis, 1851) | Javan Myna | Grassland, Wetlands | Omnivore | 2 | 0 | 2 | 0 |
| *Arachnothera longirostra*  (Latham, 1790) | Little Spiderhunter | Forest, Shrubland, | Omnivore | 0 | 0 | 0 | 2 |
| *Hierococcyx fugax*  (Horsfield, 1821) | Malaysian Hawk Cuckoo | Forest | Insectivore | 0 | 0 | 0 | 1 |
| *Rhipidura javanica*  (Sparrman, 1788) | Malaysian Pied Fantail | Forest, shrubland, mangrove, plantations | Insectivore | 0 | 0 | 2 | 0 |
| *Blythipicus rubiginosus*  (Swainson, 1837) | Maroon Woodpecker | Forest, Wetlands | Insectivore | 0 | 0 | 0 | 2 |
| *Malacopteron magnirostre*  (Moore, 1854) | Moustached Babbler | Forest, Shrubland, | Insectivore | 0 | 0 | 0 | 1 |
| *Pycnonotus plumosus*  (Blyth, 1845) | Olive-winged Bulbul | Forest, Shrubland, Grassland, | Omnivore, frugivore, insectivore | 0 | 0 | 2 | 4 |
| *Cinnyris jugularis*  (Linnaeus, 1766) | Olive-backed Sunbird | Forest, Shrubland, Grassland, | Frugivore, insectivore | 0 | 0 | 0 | 1 |
| *Dicaeum trigonostigma*  (Scopoli, 1786) | Orange-bellied Flowerpecker | Forest, Shrubland, | Frugivore, insectivore | 0 | 0 | 0 | 0 |
| *Copsychus saularis*  (Linnaeus, 1758) | Oriental Magpie Robin | Forest, Shrubland, Wetlands | Insectivore | 4 | 0 | 3 | 2 |
| *Treron vernans*  (Linnaeus, 1771) | Pink-necked Green Pigeon | Forest, | Frugivore | 0 | 0 | 1 | 0 |
| *Hypogramma hypogrammicum*  (S.Muller, 1843) | Purple-naped Spiderhunter | Forest, Wetlands | Nectarivore, frugivore, insectivore | 0 | 0 | 0 | 4 |
| *Nyctyornis amictus*  (Temminck, 1824) | Red-bearded Bee-Eater | Forest, Wetlands | Insectivore | 0 | 0 | 0 | 2 |
| *Pycnonotus brunneus*  (Blyth, 1845) | Red-eyed Bulbul | Forest, Shrubland, Wetlands | Frugivore | 0 | 0 | 0 | 1 |
| *Lonchura punctulate*  (Linnaeus, 1758) | Scaly Breasted Munia | Forest, Shrubland, Grassland, | Granivore | 0 | 0 | 1 | 0 |
| *Dicaeum cruentatum*  (Linnaeus, 1758) | Scarlet Backed Flowerpecker | Forest | Frugivore | 0 | 0 | 1 | 0 |
| *Caloramphus hayii*  (J.E.Gray, 1831) | Sooty Barbet | Forest | Frugivore | 0 | 0 | 0 | 1 |
| *Luscinia cyane*  (Pallas, 1776) | Siberian Blue Robin | Forest, Shrubland, | Insectivore, omnivore | 0 | 0 | 0 | 2 |
| *Pycnonotus erythropthalmos*  (Hume, 1878) | Spectacled Bulbul | Forest, Wetlands | Omnivore | 0 | 0 | 0 | 3 |
| *Pelargopsis capensis*  (Linnaeus, 1766) | Stork-billed Kingfisher | Forest, Wetlands | Piscivore | 0 | 0 | 1 | 0 |
| *Pycnonotus finlaysoni*  (Strickland, 1844) | Stripe-throated Bulbul | Forest, Shrubland, Wetlands | Frugivore, insectivore | 1 | 2 | 0 | 1 |
| *Copsychus malabaricus*  (Scopoli, 1786) | White-rumped Shama | Forest, Shrubland | Insectivore | 0 | 0 | 1 | 4 |
| *Amaurornis phoenicurus*  (Pennant, 1769) | White-breasted Waterhen | Forest, Shrubland, Grassland, Wetlands | Granivore, insectivore | 1 | 0 | 1 | 0 |
| *Halcyon smyrnensis*  (Linnaeus, 1758) | White-throated Kingfisher | Forest, Wetlands | Carnivore | 10 | 0 | 3 | 0 |
| *Alophoixus phaeocephalus*  (Hartlaub, 1844) | Yellow-bellied Bulbul | Forest | Omnivore | 0 | 0 | 0 | 1 |
| *Pycnonotus goiavier*  (Scopoli, 1786) | Yellow-vented Bulbul | Forest, Shrubland, Wetlands | Frugivore, insectivore | 4 | 2 | 4 | 1 |
| *Dicaeum chrysorrheum*  (Temminck, 1829) | Yellow-vented Flowerpecker | Forest | Frugivore | 0 | 0 | 0 | 1 |
| *Geopelia striata*  (Linnaeus, 1766) | Zebra Dove | Shrubland | Omnivore | 2 | 0 | 5 | 1 |

Table S2: Shannon diversity (*H’*) of understory birds across four habitat types. *H’* accounts for both species richness and evenness, with higher values indicating greater diversity.

| Habitat Type | Shannon Diversity (*H’*) |
| --- | --- |
| Forest | 2.75 |
| Oil palm | 3.06 |
| Orchard | 2.80 |
| Rubber tree | 1.10 |


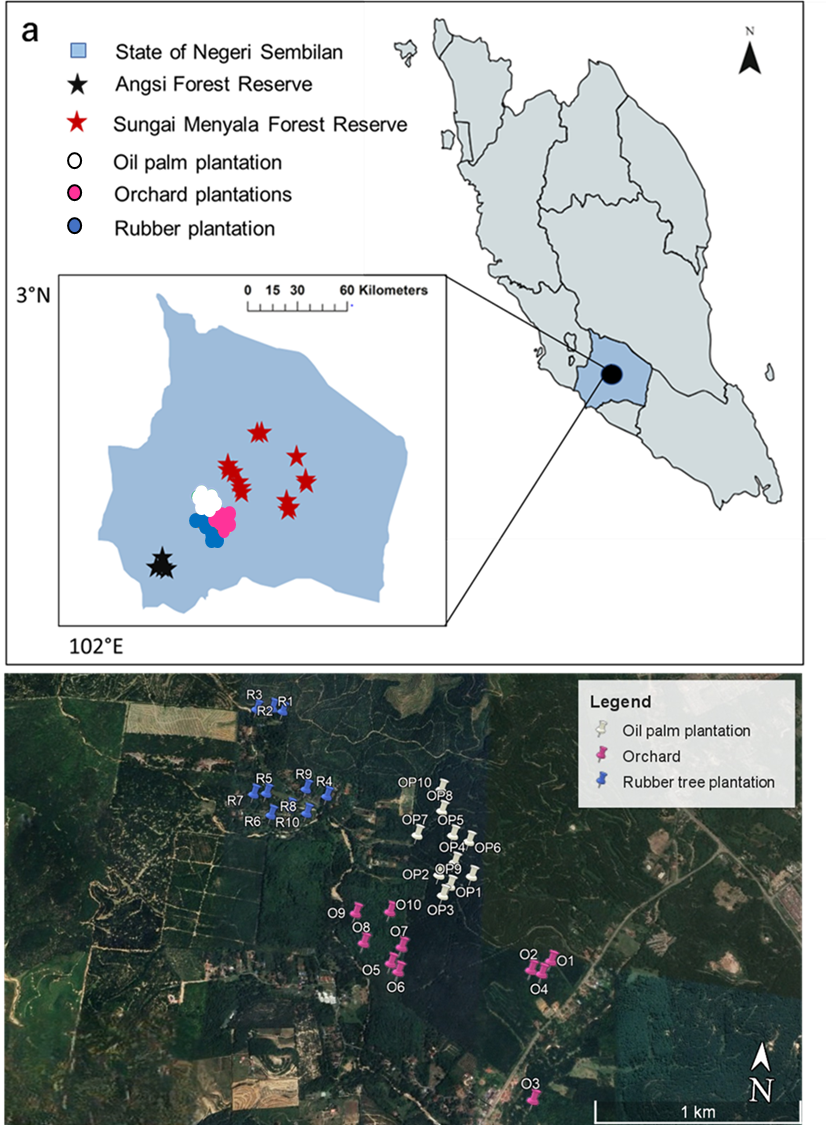


Figure S1. Map of the sampling location in plantations and forest reserves in Negeri Sembilan, Malaysia (Zoom-out (a) and zoom-in (b)).


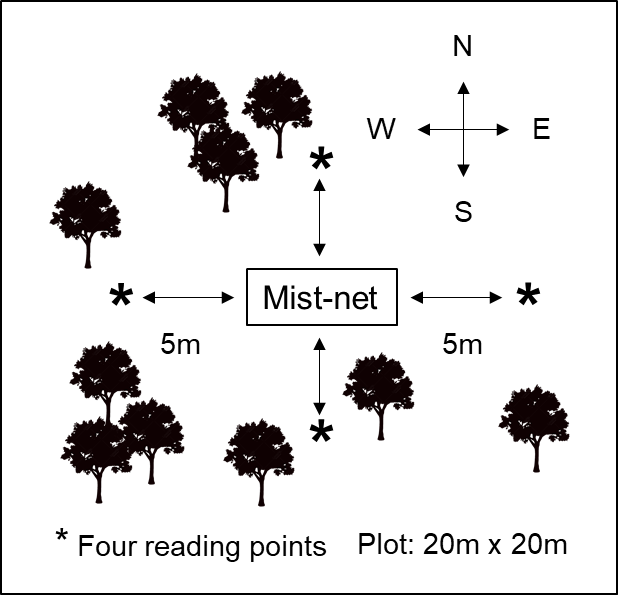


Figure S2. Schematic layout of mist-net in the sampling point location within 20 m x 20 m plot (trees shown in diagram are for illustration purposes only; actual tree locations varied).

Figure S3: Percentage of bird feeding guild in four different landscapes; Orchards, oil palms, rubber tree plantations and forest


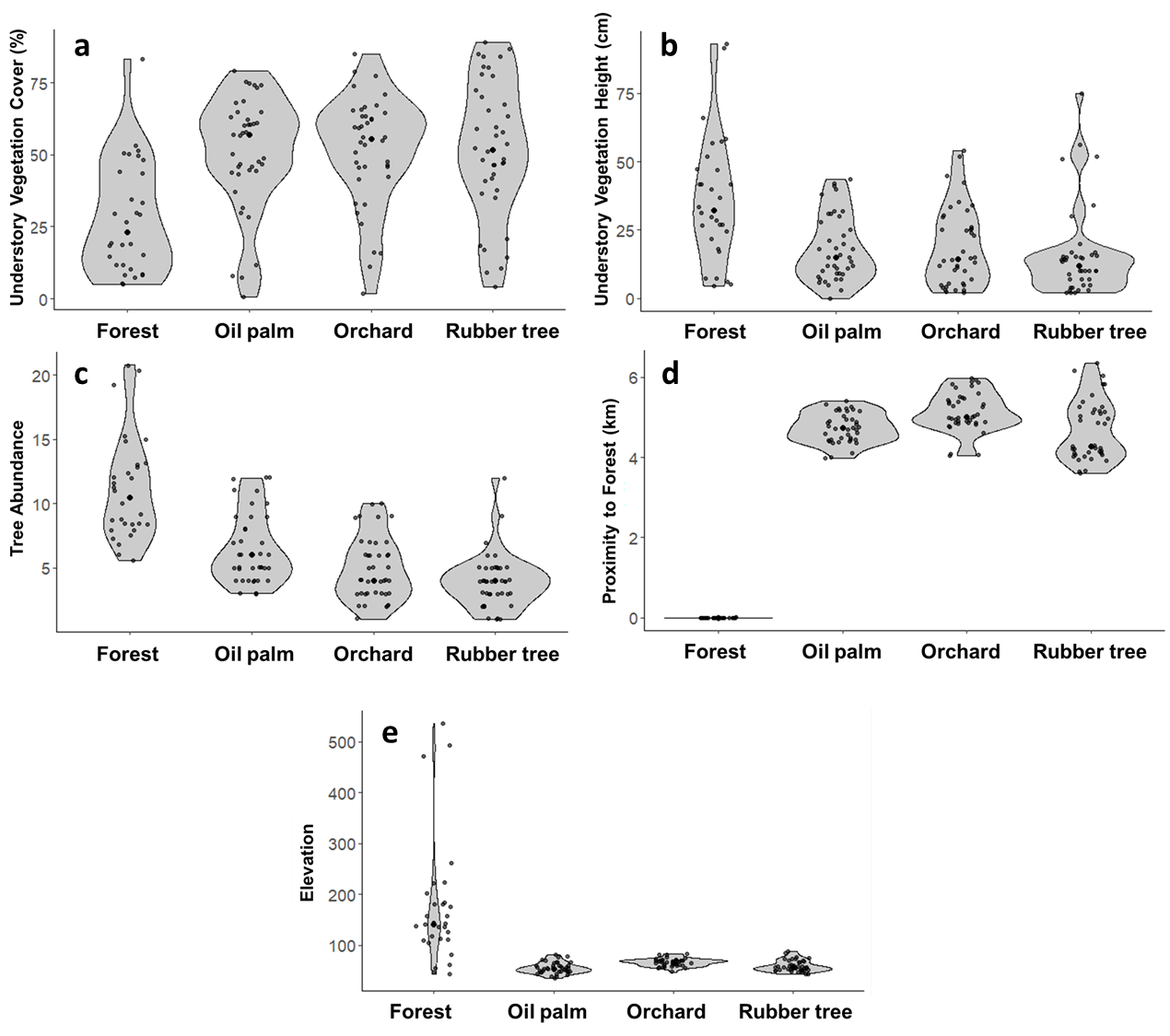


Figure S4: Violin plot of (a) understory vegetation cover (%), (b) understory vegetation height (cm), (c) mature tree abundance, (d) elevation (m), (e) proximity to forest (km) for each habitat type.
